# On the Role of Sensorimotor Experience in Facial Expression Perception

**DOI:** 10.1162/jocn_a_02148

**Published:** 2024-12-01

**Authors:** Shruti Japee

**Affiliations:** National Institute of Mental Health

## Abstract

Humans recognize the facial expressions of others rapidly and effortlessly. Although much is known about how we perceive expressions, the role of facial experience in shaping this remarkable ability remains unclear. Is our perception of expressions linked to how we ourselves make facial expressions? Are we better at recognizing other's facial expressions if we are experts at making the same expressions ourselves? And if we could not make facial expressions at all, would it impact our ability to recognize others' facial expressions? The current article aims to examine these questions by explicating the link between facial experience and facial expression recognition. It includes a comprehensive appraisal of the related literature and examines three main theories that posit a connection between making and recognizing facial expressions. First, recent studies in individuals with Moebius syndrome support the role of facial ability (i.e., the ability to move one's face to make facial expressions) in facial expression recognition. Second, motor simulation theory suggests that humans recognize others' facial expressions by covertly mimicking the observed expression (without overt motor action) and that this facial mimicry helps us identify and feel the associated emotion. Finally, the facial feedback hypothesis provides a framework for enhanced emotional experience via proprioceptive feedback from facial muscles when mimicking a viewed facial expression. Evidence for and against these theories is presented as well as some considerations and outstanding questions for future research studies investigating the role of facial experience in facial expression perception.

## INTRODUCTION

The ability of humans to extract meaningful information from the faces of others during interpersonal exchanges is a fundamental feature of social interactions. It allows for successful communication between individuals and forms the basis of interpersonal relationships. Numerous studies have examined the behavioral and neural correlates of facial expression perception including many by Leslie Ungerleider herself in both humans (Pitcher, Japee, Rauth, & Ungerleider, 2017; Zhang et al., 2016; Japee, Crocker, Carver, Pessoa, & Ungerleider, 2009; Pessoa, Japee, Sturman, & Ungerleider, 2006; Pessoa, Japee, & Ungerleider, 2005) and nonhuman primates (Zhang et al., 2023; Zhang, Japee, Stacy, Flessert, & Ungerleider, 2020; Hadj-Bouziane, Bell, Knusten, Ungerleider, & Tootell, 2008). An emerging view regarding facial expression perception is that it may rely on specialized neural circuitry (Pitcher & Ungerleider, 2021; Pitcher et al., 2017) that is distinct from other aspects of face processing such as facial identity (Ding & Zhang, 2022) and orientation (Taubert et al., 2020). Together, these studies underscore the importance of this remarkable ability of humans to extract meaningful information from facial expressions that is useful for interpersonal interactions.

However, how do we come to have this unique and well-honed ability to continuously decode a person's facial expression and combine it with other communication information such as speech and body language? Why are some people better at identifying facial expressions than others? What aspects lead to improved “reading” of social and emotional states from facial expressions? One logical answer to these questions is experience, which can in this context take one of two forms. First, the more experience you have in viewing others' facial expressions the better you might be at detecting and recognizing facial expressions. This is the classic practice effect and can be termed as *visual facial experience*—that is, experience with viewing and recognizing facial expressions and by extension, identifying the underlying emotions that the person is trying to express. Like with all things, the more you do this, the better you get. Unsurprisingly, increased visual facial experience has been shown to be linked to better facial expression recognition (Schoeneman Patel et al., 2022; Dollinger et al., 2021) and this aspect has been used in social cognition training to improve interpersonal interactions (Trujillo et al., 2017). Although a couple of studies in congenitally blind individuals (Matsumoto & Willingham, 2009; Peleg et al., 2009) have previously reported that the production of spontaneous facial expressions is innate and does not depend on the ability to see others making facial expressions, other studies have shown that increased early experience with emotional facial displays (especially of anger) may enhance sensitivity to facial expressions (Pollak & Sinha, 2002). Thus, facial expression perception is amenable to expertise and practice could improve one's visual sensitivity to expressive facial displays.

**Figure 1.**
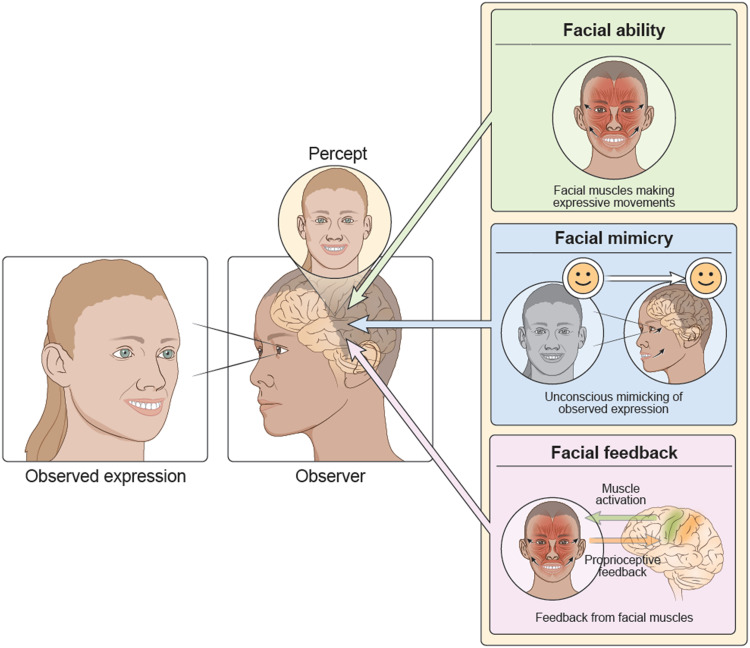
Schematic depicting the role of sensorimotor experience in facial expression perception. The three core components of sensorimotor facial experience, namely, facial ability (the ability to move one's facial muscles to make facial expressions), facial mimicry (the unconscious mimicking of a viewed facial expression), and facial feedback (the proprioceptive feedback from facial muscles), together influence the percept of the facial expression being viewed by the observer.

The second aspect of experience (and the focus of this article) that can influence how we learn to recognize facial expressions is the act of making expressions ourselves. This type of facial experience, referred to here as *sensorimotor facial experience*, is composed of three different components (see Figure 1). The purpose of this perspective article is to unpack the potential role and importance of each of these components and their contribution to facial expression perception.

### Sensorimotor Facial Experience Defined

Consider two infants—identical twins—who come into this world with similar genetic material. Assume that the parents interact with rich facial expressions with one twin but not with the other. How would this affect the ability of the two twins to recognize facial expressions? It seems likely that the first twin with greater visual exposure to facial expressions, that is, the twin with greater visual facial experience, will do better. Now consider for a moment if the two infants received similar visual experience with facial expressions (i.e., parents and others around the infants interacted to the same degree with the twins with similar amounts of facial expression use), but one twin is allowed to freely move their facial muscles, whereas the other is unable to or is restricted from moving the muscles of their face. In this scenario, would the two twins show the same level of facial expression recognition? One possibility is that the twin with the ability to move their facial muscles, that is, the twin with greater sensorimotor facial experience, would be better at recognizing others' facial expressions. First, the act of moving one's facial muscles (*facial ability*) might itself influence how those same actions are perceived in others. Furthermore, the first twin would be able to mimic their parents smile or grimace (human version of monkey see, monkey do)—a process termed *facial mimicry*—which is thought to be one way that humans learn to make facial expressions during development. And finally, the motor movements might entrain the somatic sensation of making the expression—a process termed *facial feedback*—that in turn could enhance the experience of making the facial expression in the twin. Sensorimotor facial experience is thus a conglomeration of a few things (see Figure 1): the physical capacity to move the facial muscles (facial ability), the process of mimicking others' expressions (facial mimicry), and the proprioceptive feedback from the muscles (facial feedback). Previous reviews on these topics (Niedenthal, Wood, Rychlowska, & Korb, 2017; Wood, Rychlowska, Korb, & Niedenthal, 2016; Niedenthal, Mermillod, Maringer, & Hess, 2010) have often focused on one aspect of sensorimotor experience, namely, embodied simulation or facial mimicry. The current article goes beyond these reviews by broadening the focus and including all aspects of facial experience that could impact facial expression perception. Although the three aspects of facial experience are tightly intertwined, this article aims to summarize studies related to these different aspects and provide a framework for future studies probing the link between sensorimotor experience with facial expression perception.

### Facial Ability and Facial Expression Perception

**Figure 2.**
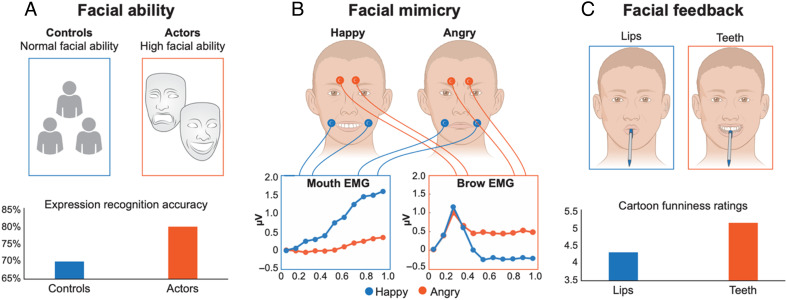
Schematic showing examples of studies from the literature investigating the three different components of sensorimotor experience. (A) Schematic of a study (Conson et al., 2013) showing increased expression recognition accuracy in actors with high facial ability (orange bar) compared with controls with average facial ability (blue bar). (B) Schematic of a facial mimicry study (Dimberg & Thunberg, 1998) showing increased muscle activity in EMG electrodes placed around the mouth when participants viewed happy faces (blue) relative to angry faces (orange) and sustained activity in electrodes placed around the brow when participants viewed angry faces (orange) but not happy faces (blue). (C) Schematic of a study (Strack et al., 1988) examining the effect of facial feedback showing greater funniness ratings of cartoons when participants held a pen between their teeth (orange) versus between their lips (blue).

Facial ability is related to the overt or explicit activation of facial muscles to make facial expressions. As with visual expertise, having more experience making a movement has been shown to lead to better tuning, detection, and recognition of the same movement in others. For example, dancers who have expertise portraying emotion with their bodies tend to be better at perceiving portrayed body expressions (Orlandi, Zani, & Proverbio, 2017; Christensen, Gomila, Gaigg, Sivarajah, & Calvo-Merino, 2016; Calvo-Merino, Glaser, Grèzes, Passingham, & Haggard, 2004). Consider the previous example of the identical twins, where both infants are able to make facial expressions from birth, but one twin undergoes intense acting training to portray expressions on their face to a greater degree than the other twin. One might predict that this twin with greater motoric experience making expressive facial movements would be able to recognize others' facial expressions better than their sibling. Indeed, professional actors trained to portray facial expressions (see Figure 2A) have been shown to have better explicit recognition of others' facial expressions (Conson et al., 2013). In fact, this link between facial expressivity and expression perception has been leveraged by researchers to use facial ability training to improve facial expression recognition in individuals with schizophrenia, where low affect or limited facial expressivity tends to be correlated with severity of schizophrenic symptoms (Pancotti et al., 2021; Popova et al., 2014). Thus, the ability to make facial expressions could play a role in shaping the recognition of expressions made by others.

### Facial Mimicry and Facial Expression Perception

Although facial ability is related to the actual motoric act of making facial expressions, the covert or implicit activation of facial muscles as seen in facial mimicry is also thought to influence facial expression perception. Babies learn the motor sequences for smiling and making other facial expressions during development (Tautermannova, 1973; Wolff, 1963). Some evidence suggests that humans learn to express emotions via facial mimicry, which can be thought of as the human version of monkey see, monkey do (Ross & Atkinson, 2020; Gallese & Sinigaglia, 2011; Keysers & Gazzola, 2009; Rizzolatti, Fogassi, & Gallese, 2001). This notion has been formulated in the literature as motor simulation theory (Jeannerod, 2001). Thus, as an infant sees its parent smile, the visual cues of the observed motion are converted into motor actions of the baby's own facial muscles (Soussignan et al., 2018; Isomura & Nakano, 2016). For example, in an electromyographic study of 5-month-old infants, researchers (Isomura & Nakano, 2016) found that the brow and cheek muscles were more engaged when the babies viewed dynamic audiovisual cries and laughter (but not unimodal stimuli), respectively. These results provided support for the notion that automatic facial mimicry is present as early as 5 months of age and can be linked to social interactions encountered in developments (e.g., parent cooing and smiling at baby). In another study using Baby-Facial Action Coding System (Oster, 2003; a version of the Facial Action Coding System [Ekman & Friesen, 1975] adapted for infant facial muscle actions) of 3-, 7-, and 12-month-olds, all infants demonstrated valence-congruent facial responses while viewing dynamic facial expressions made by a virtual model (Soussignan et al., 2018). In another study (Dimberg & Thunberg, 1998), researchers measured the facial reactions of adult participants using electromyography and found automatic and subtle movement of the zygomatic major and corrugator supercilii muscles when viewing happy and angry face images, respectively (see Figure 2B). This subtle movement occurred rapidly (around 300–400 msec) after stimulus presentation suggesting that these involuntary facial reactions are likely generated by biologically controlled fast operating “motor programs” that mimic the viewed expression. Similar results have been reported by other groups as well for different types of smiles (Korb, With, Niedenthal, Kaiser, & Grandjean, 2014; Rychlowska et al., 2014; for detailed reviews of this literature, see Niedenthal et al., 2017; Wood, Rychlowska, et al., 2016; Niedenthal et al., 2010). It has been suggested that this spontaneous mimicry may reflect an internal simulation of the perceived emotion that may further facilitate its understanding and improve empathy (Prochazkova & Kret, 2017). Thus, it would be natural to ask whether this automatic mimicry of viewed facial expressions affects the recognition of facial expressions. Specifically, does disruption or enhancement of facial mimicry impact facial expression perception?

Several studies have examined the facilitative effect of facial mimicry on facial expression recognition. A common method used to investigate this involves blocking facial mimicry from occurring and measuring its effect on recognition accuracy. In a classic mimicry blocking study (Oberman, Winkielman, & Ramachandran, 2007), researchers used two different mimicry interference methods by asking participants to either bite on a pen or chew gum, while measuring EMG over the cheek, mouth, and nose regions. They found that the bite manipulation interfered consistently with the recognition of happiness whereas the chewing manipulation affected the recognition of angry expressions. Another study tested the ability of participants to identify facial expressions while disrupting their ability to engage in facial mimicry by using a pen-bite manipulation, and found that blocking mimicry affected expression recognition performance but not gender discrimination (Borgomaneri, Bolloni, Sessa, & Avenanti, 2020). Similar results of reduced expression recognition performance have also been reported by others in studies where facial mimicry was physically disrupted (Wood, Lupyan, Sherrin, & Niedenthal, 2016). Approaching it from a different angle, another study asked participants to pose a happy, disgusted, or neutral facial expression and then asked them to watch an ambiguous face (morph between happiness and disgust) and indicate whether the emotion perceived was happiness or disgust (Benuzzi et al., 2023). They found that posing an emotional face increased the percentage of congruence with the perceived emotion. These studies together provide support for the idea that facial mimicry contributes to the recognition of facial expressions.

### Facial Feedback and Facial Expression Perception

Charles Darwin noted in his 1872 work on *The expression of the emotions in man and animals* that if a facial expression is allowed to be made freely, its experience is intensified (Darwin, 1872). Conversely, if an expression is repressed, its associated emotion is softened. Darwin's postulation was furthered by William James and Carl Lange (Lange, 1885; James, 1884) who linked the proprioceptive feeling of making an expression (facial feedback) with the emotion itself. Empirical work to test these theories has led to much debate in the literature about the strength of the facial feedback hypothesis (with weak and strong versions proposed). A seminal study that tested this hypothesis used facial manipulations that either induced a smile or a frown (by asking participants to hold a pen between their teeth or between their lips, respectively) and showed a significant modulation of humor ratings of cartoons (Strack, Martin, & Stepper, 1988). Despite its prominence in the literature and support from other studies (de la Rosa, Fademrecht, Bulthoff, Giese, & Curio, 2018; Goldman & Sripada, 2005; Soussignan, 2002), a recent large-scale registered report across 17 independent research groups failed to replicate the original finding, thereby casting doubt on the validity of the feedback hypothesis (Wagenmakers et al., 2016). Although a more recent large-scale meta-analysis provides support for the idea that facial feedback does influence emotional experience, albeit in a small and heterogenous way (Coles, Larsen, & Lench, 2019), more work is needed to resolve this debate. Thus, future research will help clarify whether proprioceptive feedback from skeletal muscles of the face plays a role in the recognition of facial expressions and the experience of the associated emotion.

### A Continuum of Sensorimotor Facial Experience

**Figure 3.**
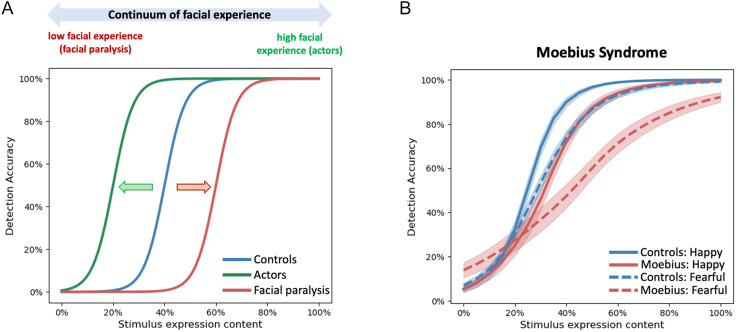
(A) Hypothesized expression detection psychometric functions for the two extreme cases of sensorimotor facial experience, facial paralysis with no ability to move one's facial muscles, and professional actors who have considerable experience making facial expressions. Relative to controls, actors would likely show a shift to the left in the psychometric function for expression detection, whereas individuals with facial paralysis would show a shift to the right. (B) Measured psychometric functions for happy (solid lines) and fearful (dashed lines) for controls (in blue) and individuals with Moebius syndrome (in red) showing a shift to the right (higher detection thresholds) for the latter (Japee et al., 2023).

Overall, the three components of sensorimotor facial experience, namely, facial ability, facial mimicry, and facial feedback, all appear to contribute toward our ability to recognize facial expressions. However, the degree to which sensorimotor experience shapes expression perception may depend on the manner in which the experience is gathered, how much of it is amassed, and when. If an infant is prevented from making facial expressions for the first few years of their life (thus delaying the age of acquisition of facial experience), they may experience lower sensitivity to subtle facial expressions. Conversely, if a child grows up making expressive and animated facial movements from birth, they may likely be better at recognizing others' facial expressions. Such developmental differences in experience acquisition could result in a continuum of facial experience that in turn might produce varying levels of impact on expression perception (see Figure 3A). For example, individuals with high facial experience such as professional actors (high end of the continuum) might show high sensitivity to low levels of facial expressions, that is, a shift in the expression detection psychometric curve to the left compared with normal individuals. On the other hand, individuals with low facial experience might show limited sensitivity to subtle facial expressions (i.e., a shift in psychometric curve to the right). Much can be understood about the overall role of sensorimotor experience in facial expression perception by disrupting the ability to make facial expressions. This can be achieved naturally (via temporary, permanent, or congenital facial paralysis) or artificially (by temporary chemical disruption of the ability to make facial expressions). The different types of disruption alter the contributions of each of the three components of sensorimotor experience to a different degree. These aspects are further explored below.

### Disruption of Sensorimotor Facial Experience

A pharmacological method that allows temporary disruption of facial experience involves the use of botulinum toxin to selectively inactivate facial muscles involved in making certain facial expressions. In a recent study, researchers using botulinum toxin injections into the “frown area” of the face (thus disrupting facial mimicry and facial feedback) demonstrated that unlike un-injected control participants who showed a pre to post RT improvement on a task involving detection of angry facial expressions, injected participants showed no such improvement (Bulnes, Marien, Vandekerckhove, & Cleeremans, 2019). Furthermore, a recent neuroimaging study (Stark, Stark, Wong, & Brin, 2023) found that preventing frowning through use of botulinum toxin resulted in a compensatory increase in amygdala activity (related to reduced facial feedback) during viewing of expressive faces. A similar study using botulinum toxin to reduce facial feedback found attenuated responses in left amygdala during imitation of angry expressions, as well as in its functional coupling to autonomic brain stem regions implicated in manifesting emotional states (Hennenlotter et al., 2009). Thus, eliminating facial mimicry and proprioceptive feedback temporarily via a pharmacological agent appears to alter perceptual behavior and neural activity underlying emotional processing. So much so that botulinum toxin injections have been proposed as a potential treatment option for depression (Wollmer, Magid, Kruger, & Finzi, 2021, 2022; Finzi & Rosenthal, 2016; Alam, Barrett, Hodapp, & Arndt, 2008).

Natural disruption of the ability to make facial expressions can be seen in cases of Bell's palsy, an idiopathic facial paralysis that renders the facial muscles on one side of the face immovable. A recent study examining facial expression recognition in Bell's palsy patients found delayed recognition compared with controls of emotional facial expressions, but not facial identities (i.e., patients had longer RTs than controls for expression recognition but not identity recognition; Storbeck, Schlegelmilch, Streitberger, Sommer, & Ploner, 2019). In another study, examining emotion recognition in stroke patients with and without facial palsy (i.e., with and without loss of facial motor function), researchers found facial expression recognition deficits only in the facially affected subgroup. Furthermore, these same patients showed no deficits in recognizing emotion from affective voices (Kuttenreich, von Piekartz, & Heim, 2022), suggesting that the deficit is related to the inability to move one's facial muscles. Another natural disruption of the ability to make facial expressions can be seen in Parkinson's disease where several studies have reported reductions in expression recognition likely because of the motor disturbances affecting facial mimicry and facial feedback (Kuehne, Polotzek, Haghikia, Zaehle, & Lobmaier, 2023; Chuang et al., 2022; Kang, Derva, Kwon, & Wallraven, 2019; Argaud et al., 2016; Livingstone, Vezer, McGarry, Lang, & Russo, 2016; for reviews, see Argaud, Vérin, Sauleau, & Grandjean, 2018; Prenger & MacDonald, 2018). Reductions in facial mimicry have also been reported in individuals with alexithymia who often show emotion recognition deficits (Franz et al., 2021; Nordmann, Schäfer, Müller, & Franz, 2021). Although all these studies provide valuable information about the link between sensorimotor facial experience and expression perception, individuals in these studies have been able to make expressions from birth and the disruption occurs later in life and thus cannot be fully controlled for other factors. Thus, a better test of the role of sensorimotor facial experience comes from cases of congenital facial paralysis such as that seen in Moebius syndrome (MBS), where individuals are unable to move their facial muscles from birth, thus disrupting all three aspects of sensorimotor facial experience—facial ability, facial mimicry, and facial feedback.

### Expression Perception in Individuals with MBS

MBS is a rare congenital disorder characterized by limited lateral gaze and non-progressive facial paralysis because of the underdevelopment or absence of the sixth (abducens) and seventh (facial) cranial nerves or nuclei. As a result, individuals with MBS are unable to smile, frown, grimace, or make other facial expressions from birth. Thus, this condition provides an ideal model to investigate how the inability to express emotions with one's face affects the ability to process and recognize facial expressions made by others.

The few early studies of individuals with MBS produced conflicting results, with some studies concluding that the inability to make facial expressions does not impact expression perception (Vannuscorps, Andres, & Caramazza, 2020; Rives Bogart & Matsumoto, 2010; Calder, Keane, Cole, Campbell, & Young, 2000), whereas others reported deficits in emotional expression recognition in individuals with MBS (De Stefani, Ardizzi, et al., 2019; De Stefani, Nicolini, Belluardo, & Ferrari, 2019; Bate, Cook, Mole, & Cole, 2013; Giannini, Tamulonis, Giannini, Loiselle, & Spirtos, 1984). However, these prior studies had critical limitations and were likely not sensitive enough to pick up any subtle impairments in facial expression perception in individuals with MBS. In a recent study, we systematically investigated the role of sensorimotor experience and, thus, facial ability, facial mimicry, and facial feedback, in facial expression perception by using a comprehensive battery of sensitive expression detection tasks in a group of MBS individuals (Japee et al., 2023). We found that individuals with MBS were impaired at detecting emotion in others' facial expressions and this impairment in expression detection was accompanied by an impairment in facial motion perception. Importantly, although the facial paralysis affected the sensitivity of MBS individuals to facial motion and facial expressions, it did not impact their ability to perceive body motion or extract emotional information from body expressions; they were also able to accurately perform other challenging tasks such as feature detection and identity matching. Thus, the inability to move a particular body part (in this case one's face) appears to impact the perception of motion and emotional information in the same body part of others (here facial expressions vs. body expressions), resulting in a selective dampening of facial expression perception. Figure 3B shows an example of the type of impairment seen in individuals with MBS. Relative to controls, MBS individuals showed a shift to the right in the psychometric curve measuring the accuracy of fearful and happy face detection. These convergent findings together suggest that the inability to make facial expressions from birth results in a dampening of perception of others' facial expressions and may be associated with changes in the level of engagement of the neural circuitry underlying emotion processing (Japee et al., 2023).

### Neural Mechanisms Underlying the Impact of Facial Experience

Although some studies have tried to characterize the effects of the different aspects of facial experience, most have approached it from a behavioral perspective by measuring the impact on facial expression recognition or on elicited physiological responses. However, the brain circuitry that facilitates the impact of facial ability, facial mimicry, and facial feedback on facial expression perception has not been well studied. There is a large and growing body of literature delineating the neural circuitry underlying facial expression perception in humans and nonhuman primates. Together, the view has emerged of a visual pathway that is specialized for facial motion (and thus facial expression) perception (Pitcher & Ungerleider, 2021; Pitcher et al., 2017; Duchaine & Yovel, 2015; Weiner & Grill-Spector, 2013) that involves facial motion sensitive regions along the STS, starting in posterior STS, proceeding anteriorly to anterior STS regions, and eventually culminating in the amygdala (Zhang et al., 2020). In addition to facial motion sensitivity, some studies have also shown sensitivity of STS regions to eye gaze (Babo-Rebelo et al., 2022). There is also some evidence from intracranial recordings suggesting a subcortical route via which the amygdala may receive rapid and direct information about facial expressions (Huijgen et al., 2015). Furthermore, there is some evidence supporting laterality in expression processing networks, such that certain expressions, such as fear, selectively activate the left occipitotemporal cortex and amygdala (Hardee, Thompson, & Puce, 2008). Meanwhile, several other neuroimaging studies (Sato et al., 2019; De Winter et al., 2015; Fusar-Poli et al., 2009) have reported right lateralized activations in response to facial expressions; thus, more work is needed to resolve the laterality debate.

Although these cortical and subcortical routes for facial expression processing have been the focus of much research, there have been only a few attempts at examining the modulation of this circuitry by facial experience. In one such attempt, Japee and colleagues recently reported preliminary findings of reduced fMRI activity in posterior STS for a small group of individuals with MBS (characterized by congenital facial paralysis) compared with a matched control group during expression matching, whereas activation in the fusiform face area did not differ between the two groups (Japee et al., 2023). In addition, there was a trend for the right amygdala to be deactivated relative to controls when MBS individuals performed an expression matching task but not identity matching. Another recent study examining functional connectivity in EEG data in individuals with congenital facial palsy (resulting from MBS) found reduced connectivity between sensorimotor and visual face processing regions in MBS individuals relative to controls (Quettier, Maffei, Gambarota, Ferrari, & Sessa, 2023). In another study, using high-density electroencephalography, MBS and control participants were found to activate different pathways during facial expression processing, with controls showing greater activity in right posterior STS and regions critical for sensorimotor simulation such as premotor areas, whereas MBS individuals showed greater engagement of ventral-temporal regions (Sessa et al., 2022). In addition, ERPs elicited by pictures of smiling faces measured after TMS over sensorimotor face regions have demonstrated involvement of these regions in judging the authenticity of the smiles (Paracampo, Tidoni, Borgomaneri, di Pellegrino, & Avenanti, 2017). Repetitive TMS over somatosensory face regions has also been shown to impair performance on a facial expression task, but not an identity task (Pitcher, Garrido, Walsh, & Duchaine, 2008), or eye gaze task (Pourtois et al., 2004), thus providing support for the simulation theory of emotion recognition (Hussey & Safford, 2009). Furthermore, using gel to block facial mimicry while measuring ERPs, researchers have shown disruptions of early stages of facial expression processing (Schiano Lomoriello, Maffei, Brigadoi, & Sessa, 2021).

Such neuroimaging and stimulation studies are crucial to understanding the impact of facial experience on facial expression processing, and although they often suffer from small sample sizes, they suggest that facial paralysis likely affects expression perception via complicated feedforward–feedback network connectivity between action, sensation, perception, and emotion processing regions. Thus, despite the sample size limitation, these studies provide clues about the neural substrates involved in the modulation of expression perception by experience. For example, one might predict that observing someone make expressions would engage regions of the action observation network such as premotor, superior-temporal, and parietal areas (Keysers & Gazzola, 2006; Rizzolatti & Craighero, 2004), which could activate implicit motors loops normally engaged in the observed expression. In addition, proprioceptive feedback from the facial muscles to somatosensory regions (Kragel & LaBar, 2016) would help encode the “feeling” of making a facial expression and engage emotion processing regions such as the amygdala. Separately, having more practice in making facial expressions, that is, better activated/engaged facial sensorimotor regions, would in turn provide modulatory input to action observation regions (Calvo-Merino, Grezes, Glaser, Passingham, & Haggard, 2006). Furthermore, one might expect that these implicated networks, that is, action, sensation, perception, and emotion processing regions, would influence each other during development to bring about the effect of facial experience in expression perception. To fully delineate the neural underpinnings of facial experience, what is needed is a series of well-powered neuroimaging studies in large groups of healthy controls (children and adults) as well as individuals with limited facial experience such as those with MBS or Bell's palsy. This will allow systematic characterization of the involvement of sensorimotor and face processing regions (including fusiform face area, posterior STS, amygdala, and frontal regions) in the facilitatory effects of facial experience.

### Outstanding Questions and Considerations for Future Research

Overall, this review provides a framework for thinking about the role of sensorimotor facial experience in facial expression perception. Whereas some research has been done to explore the contribution of the three components of sensorimotor experience, namely, facial ability, facial mimicry, and facial feedback, much work still needs to be done to overcome some of the limitations of previous studies and resolve the conflicting results in the literature. Several outstanding questions and avenues of future research outlined here could clarify the impact of experience of expression perception.

First, many of the original studies of facial mimicry and feedback employed a limited number of participants, which could in part be the reason for the conflicting results. In addition, some of the studies did not include appropriate control conditions to serve as a baseline for comparison. To alleviate this issue, multiple, large-scale replication studies with properly designed control tasks could be conducted to resolve the debate in the literature.

Second, as mentioned above, if congenital facial paralysis (as seen in MBS) serves as one end of the continuum of sensorimotor facial experience, then having extensive experience in making facial expressions, such as that seen in professional actors, could be considered as the other end of that spectrum (see Figure 3A). Thus, a future study could test the hypothesis that professional actors would show a shift to the left in their psychometric curves for expression detection, suggesting greater sensitivity to low levels of facial expression information. In addition, future studies could explore the effectiveness of visual training paradigms to enhance facial expression perception in situations involving motoric absence, such as the congenital facial palsy seen in MBS. Furthermore, although some attempts have been made recently to obtain neuroimaging data in individuals with MBS (Japee et al., 2023), future studies with larger samples sizes could provide valuable information about the neural mechanisms that link sensorimotor experience and facial expression perception.

Third, it is well known that facial expression perception is influenced by many factors, such as anxiety states and personality measures, and previous research has focused on these aspects (Kang, Kim, Kim, & Lee, 2019; Knyazev, Bocharov, Levin, Savostyanov, & Slobodskoj-Plusnin, 2008). However, facial experience as a factor impacting facial expression perception has not received much consideration. Despite its potential impact on how quickly participants may detect and recognize facial expressions, most studies do not take an individual's level of facial experience into consideration, often because of the challenges in quantifying it. Myriad factors influence facial experience, and currently, there is no straightforward way to measure them. One way to simplify this complex problem is to consider two separate sets of factors that influence facial experience—those related to the domain of action, that is, the amount of experience making facial expressions, and those related to the domain of visual consumption, that is, the amount of experience one has viewing facial expressions.

Within the action domain, by employing a frequency-of-use scale (e.g., never or not at all, rarely or a bit, occasionally or some, often or a lot, very often or a great deal), one could quantify the level of involvement in recreational and professional acting at different stages in life (e.g., during preschool years, during elementary, middle and high school, during different stages of adulthood such as during young, middle, and older adulthood). Such acting experience could be obtained in different types of performing arts such as live theater, television/film, commercials, street or pop-up theater, social media, informal workshops, and training, and so forth. Similarly, one could ask questions aimed at quantifying the level of interaction with children either at home or outside the home. For example, elementary and preschool teachers may interact with children in a more exaggerated way and thus would have more experience making intense facial expressions. This information could be combined with objective quantification via facial electromyography of the level of facial action either during facial mimicry or when explicitly making facial expressions.

On the visual consumption side, one could develop questionnaires to enquire about how much time is spent watching different types of media such as television series, cartoons, soap operas, reality television shows, movies, and so forth. This information could be gathered for different stages of life to obtain a holistic picture of the types of visual facial experience one may have engaged in during the participant's life span. In addition, questions aimed at understanding consumption of YouTube videos, social media such as Instagram, TikTok, and so forth would also be valuable. Furthermore, information about the level of engagement in watching team and individual sports, combative sports (e.g., wrestling), and expressive sports (e.g., ice skating and gymnastics) would also be useful in estimating the amount of visual facial experience, that is, experience in viewing facial expressions. The action and consumption data obtained above could then be combined to create an estimate of the total amount of facial experience and used as a covariate when studying facial expression perception in healthy controls. The utility of such questionnaires has recently been demonstrated in studies investigating the role of experience in facial identity recognition (Carter, Andrews, Japee, & Ritchie, 2023; Andrews, Japee, & Ritchie, 2022) and could be adapted to understand the impact of experience on facial expression perception as well.

Another future avenue of research could examine the impact of the lack of visual experience (as seen in congenitally blind individuals) on sensorimotor experience. Considering the example of twins, if one was born blind and could never see a facial expression, what impact would that have on their ability to make facial expressions themselves? Although a couple of studies in congenitally blind individuals (Matsumoto & Willingham, 2009; Peleg et al., 2009) have previously shown that the production of spontaneous facial expressions does not depend on the ability to see others making facial expressions, future work is needed to fully understand the effect of the absence of visual input on facial expression production. Furthermore, micro-contractions of facial muscles have been recorded in individuals with blindsight who are unable to consciously see facial and body expression stimuli presented in their blind field but appear to still respond involuntarily to them (Tamietto et al., 2009). Thus, systematic characterization of spontaneous and voluntary facial expression production in both congenitally blind and blindsight individuals could shed light on the link between observing and making facial actions. This would in turn help elucidate the basic principles of sensorimotor experience (especially facial mimicry) and their role in perception in sighted individuals.

Future work could also examine whether the impact of sensorimotor experience varies across different facial expressions and different modalities. On the basis of differential innervation patterns of the face by pyramidal and extrapyramidal upper motor-neuron tracts (de Oliveira-Souza, 2012; the pyramidal tract is thought to provide slow and voluntary innervation to the face whereas the extrapyramidal tract provides rapid, involuntary signals, more so to the lower part of the face), one might predict differential impacts of various facial expressions on facial ability, mimicry, and feedback, depending on the relative involvement of upper or lower parts of the face. Motion analysis of facial expressions suggests that a smile typically involves elongation of the mouth and some narrowing of the eyes, whereas fear involves widening of the eyes, changes in the brow area, and opening of the mouth (Huijgen et al., 2015). Meanwhile, eye tracking studies of facial expression perception have shown that participants typically look at the mouth for happy expressions and at the eyes for fearful expressions (Bodenschatz, Kersting, & Suslow, 2019; Schurgin et al., 2014; Smith, Cottrell, Gosselin, & Schyns, 2005). Thus, more work is needed to understand the impact of these differences between expressions on how facial experience impacts facial expression perception. Another avenue of future work could examine the difference between the impact of bilateral and unilateral palsy on facial expression perception. Distinguishing these various components of facial experience in this way could potentially also help clarify the conflicting results in the literature and shed light on the laterality debate. Furthermore, comparing the role of experience in perception of emotion from faces relative to other modalities such as voice and touch (for a review, see Schirmer & Adolphs, 2017) would also provide valuable information about how emotion processing is shaped by experience.

Taken together, studies investigating the role of sensorimotor experience in facial expression perception are not only important in the understanding of how humans process facial expressions, but in the wider understanding of face perception as well. Recent neuroimaging studies in human and nonhuman primates have suggested separate neural pathways subserving facial identity and facial expression processing (Pitcher & Ungerleider, 2021; Taubert et al., 2020). By combining results from studies in disorders such as MBS, Bell's palsy, Parkinson's disease, and so forth, with those from studies in other disorders of face perception such as developmental prosopagnosia (where the ability to recognize faces is missing from birth), we can begin to shed light on the neurocircuitry underlying perceptual behavior as it relates to faces, whether it be extraction of facial information to identify who a person is or what emotion they are expressing.

## Acknowledgments

Many thanks to Jessica Taubert, Maryam Vaziri-Pashkam, and Chris Baker for providing feedback on earlier versions of this article and to Ethan Tyler, NIH Medical Arts, for help with the figures. Some of the work presented in the article was conducted under Dr. Leslie G. Ungerleider's mentorship. Leslie was deeply interested in the role of action experience in perception and was intrigued by the data obtained in individuals with MBS. This article is dedicated to her memory and to her enduring influence on the field of cognitive neuroscience.

Corresponding author: Shruti Japee, 10 Center Drive, Room 4C207, Laboratory of Brain and Cognition, National Institute of Mental Health, NIH, Bethesda, MD 20892, or via e-mail: japees@mail.nih.gov.

## Data Availability Statement 

Data and analysis scripts are available upon request.

## Author Contributions

Shruti Japee: Conceptualization; Writing—Original draft.

## Funding Information

Shruti Japee is supported by the Intramural Research Program of the National Institute of Mental Health, grant number: ZIAMH002909.

## Diversity in Citation Practices

Retrospective analysis of the citations in every article published in this journal from 2010 to 2021 reveals a persistent pattern of gender imbalance: Although the proportions of authorship teams (categorized by estimated gender identification of first author/last author) publishing in the *Journal of Cognitive Neuroscience* (*JoCN*) during this period were M(an)/M = .407, W(oman)/M = .32, M/W = .115, and W/W = .159, the comparable proportions for the articles that these authorship teams cited were M/M = .549, W/M = .257, M/W = .109, and W/W = .085 (Postle and Fulvio, *JoCN*, 34:1, pp. 1–3). Consequently, *JoCN* encourages all authors to consider gender balance explicitly when selecting which articles to cite and gives them the opportunity to report their article's gender citation balance. The authors of this paper report its proportions of citations by gender category to be: M/M = .319; W/M = .253; M/W = .176; W/W = .253.
